# Massive Pulmonary Arteriovenous Malformation as a Cause of Fetal Heart Failure

**DOI:** 10.7759/cureus.52549

**Published:** 2024-01-19

**Authors:** Joao Oliveira Dias, Ana Catarina Lai, Orlando Rodrigues, Paula Martins, Miguel Branco, Raquel Pina, António Pires

**Affiliations:** 1 Pediatric Cardiology Department, Centro Hospitalar e Universitário de Coimbra, Coimbra, PRT; 2 Pathology Department, Centro Hospitalar e Universitário de Coimbra, Coimbra, PRT; 3 Genetics Department, Centro Hospitalar e Universitário de Coimbra, Coimbra, PRT; 4 Obstetrics Department, Centro Hospitalar e Universitário de Coimbra, Coimbra, PRT

**Keywords:** arteriovenous malformations, termination of pregnancy, fetal echocardiogram, cardiomegaly, fetal heart failure

## Abstract

Pulmonary arteriovenous malformations (AVMs) are abnormal connections between the pulmonary arteries and veins that can result in rapid-onset heart failure.

We present a case of a fetus with pulmonary AVMs diagnosed at 22 weeks gestation. Fetal echocardiography showed cardiomegaly and dilated pulmonary arteries and veins reflecting the hemodynamic significance of the shunt. Inverted flow through the ductus arteriosus was also present.

Fetal autopsy following medical termination of the pregnancy confirmed the morphological findings, including displacement of arteries and veins in proximity to the pleural surface. The genetic study was negative.

This report highlights the cardiovascular impact of a rare disorder. Inverted flow through the ductus arteriosus may be another poor prognostic indicator, useful in parental counseling.

## Introduction

Pulmonary arteriovenous malformations (AVMs) are rare abnormal connections between the pulmonary arteries and veins [1]. These malformations bypass the pulmonary capillaries, resulting in a right-to-left shunt. Rarely, with larger lesions, pulmonary AVM can cause cyanosis in the newborn and high-output heart failure in the early fetal period [2].

Reported mortality in cases with early presentation is high (30-40%), particularly when associated with the Osler-Weber-Rendu syndrome [3]. Reported cases of this entity during the fetal period are rare [4-8].

This case report intends to highlight the morphological findings of pulmonary AVMs and their potential impact on fetal hemodynamics.

## Case presentation

We report a case of a 35-year-old pregnant woman, referred at 22 weeks gestation due to cardiomegaly and suspected vascular lung malformations. The parents were second-degree cousins and there was a familiar history of a cerebral aneurysm (uncle) and recurrent epistaxis.

**Figure 1:**
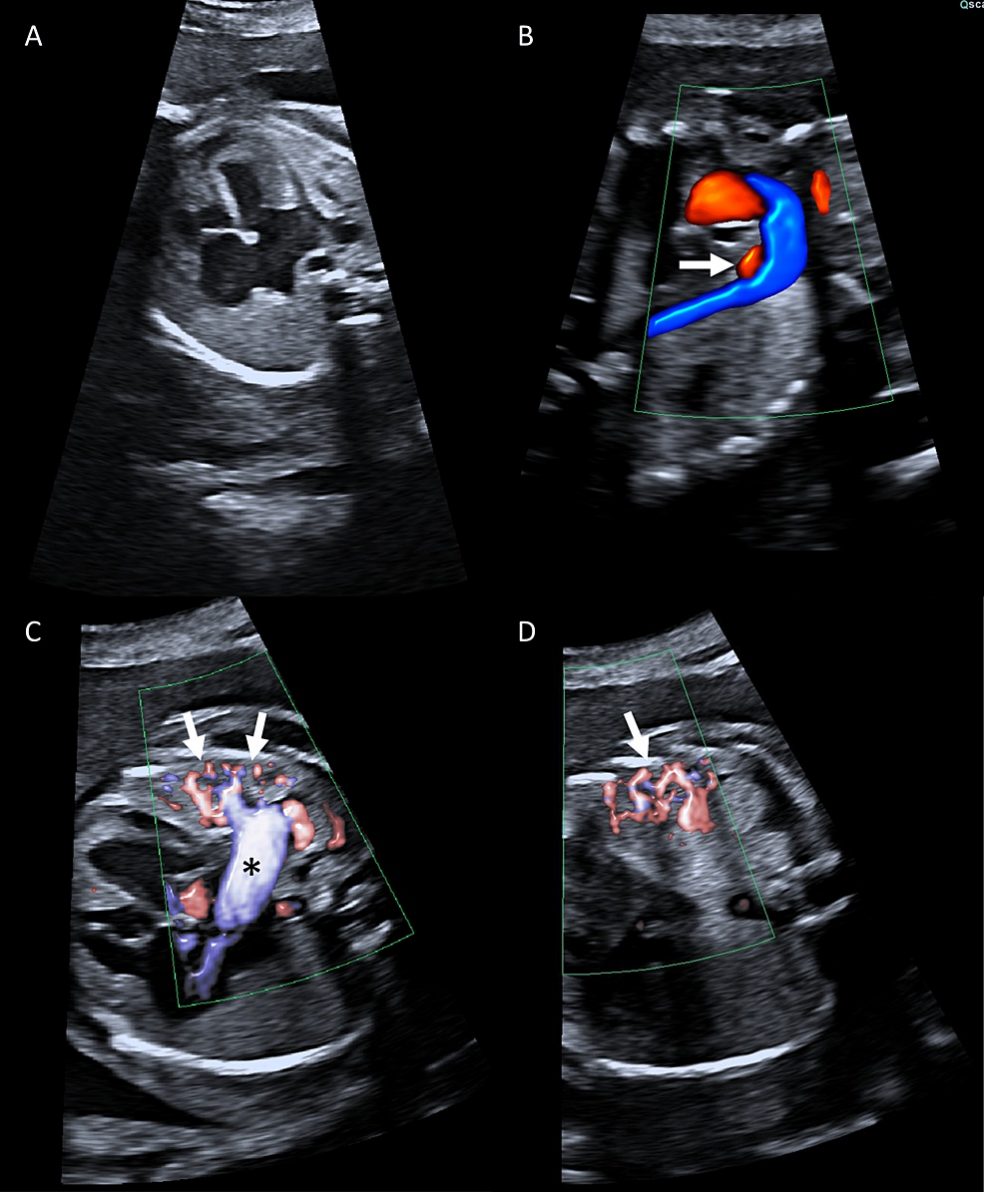
Pulmonary arteriovenous malformations in fetal echocardiogram. Fetal echocardiogram significantly increased cardiothoracic circumference ratio and biventricular hypertrophy (A), left to right shunting through the ductus arteriosus (B, arrow), enlarged left pulmonary vein (C, asterisk), and vascular malformations in the juxtaposed left lung (C and D, arrows).

A fetal echocardiogram confirmed cardiomegaly (Figure 1A) and severe left pulmonary artery and veins dilation (Figure 1B) secondary to a large AVM located in the lower left lung (Figure 1C). The hemodynamic impact of the shunt was also evident by the presence of reversed flow through the ductus arteriosus (Figure 1D).

**Figure 2:**
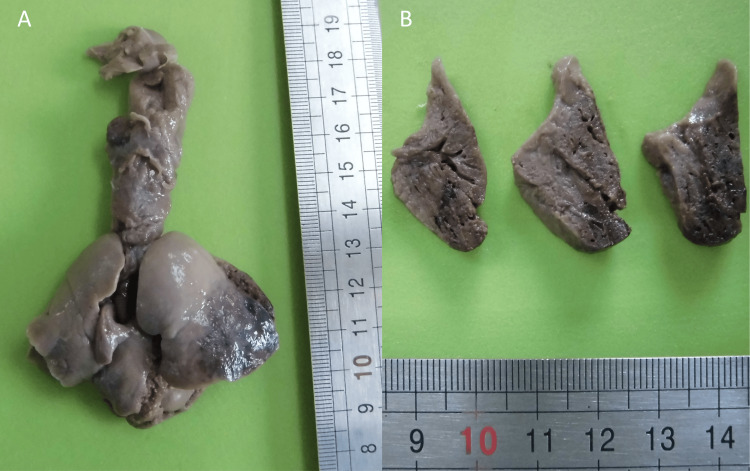
Pulmonary arteriovenous malformation fetal macroscopic anatomopathological study. Macroscopic image showing hypervascularized lower left lung (A) and cross-section of the left lung showing enlarged subpleural vascular structures (B).

**Figure 3:**
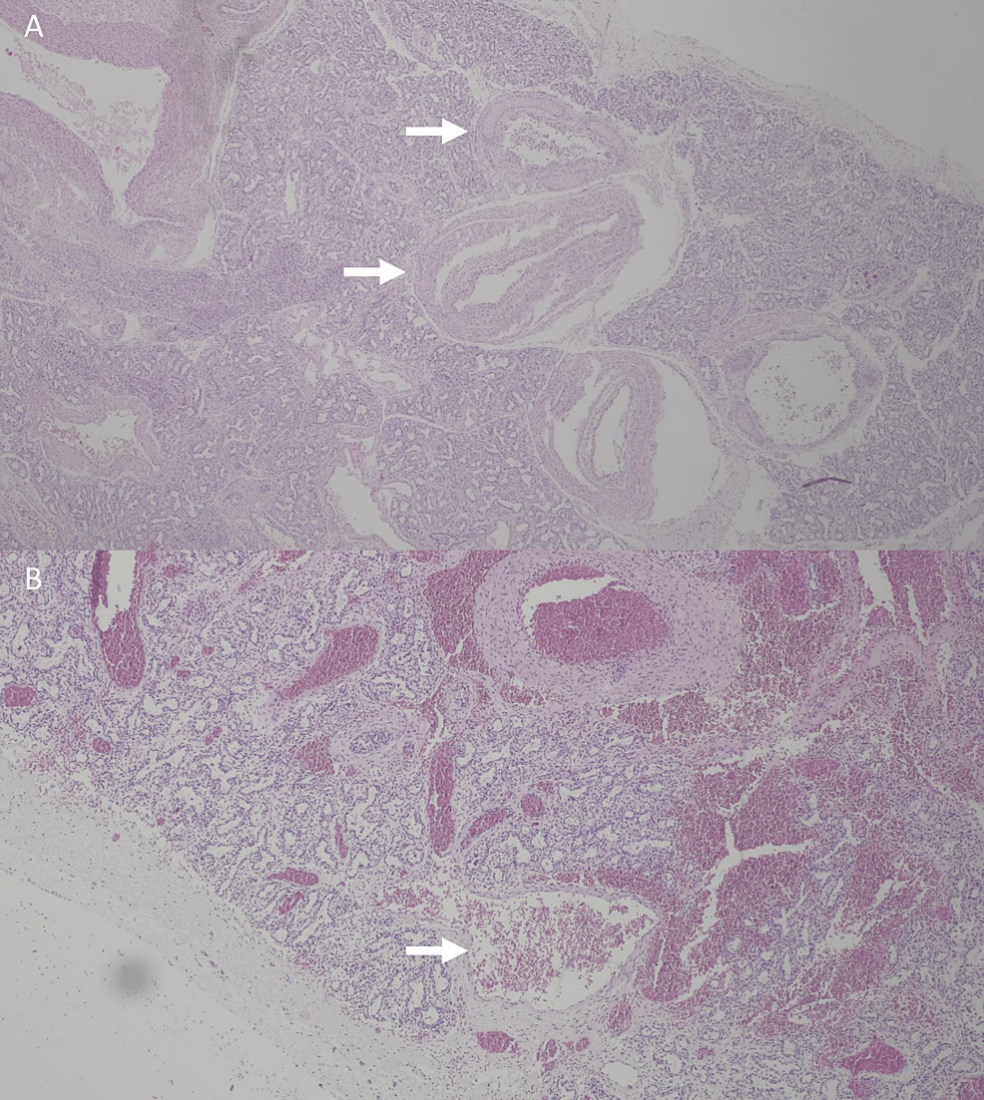
Fetal arteriovenous malformation microscopic anatomopathological study. Microscopic features of the left lobe with large and contoured arteries (A, arrow) and veins (B, arrow) in proximity to the pleural surface.

After counseling, the parents opted for the termination of the pregnancy. The autopsy showed left lower lobe vascular congestion and dilated vessels (left pulmonary artery, left inferior vein, and intra-pulmonary branches) and confirmed the cardiomegaly (Figure 2). The histologic study documented the existence of large arteries and veins, with contoured walls and irregular thickness, in continuity with each other. These vessels were also displaced in proximity to the pleural surface (Figure 3). These features were absent in the contralateral lung.

Fetal genetic testing through a next-generation sequencing panel with copy number variation analysis aimed at vascular malformations failed to identify pathogenic variants.

## Discussion

Primary pulmonary AVMs are a rare congenital defect, presenting more often in the lower lobes. The clinical spectrum is varied and depends on lesion size, ranging from asymptomatic patients, presenting in adult life, to more severe cases that may rarely present in the fetal period [3]. Even though they can occur sporadically, up to 70% of cases are associated with Osler-Weber-Rendu syndrome [1].

Treatment options include catheterization and surgery. Percutaneous device embolization can be considered for focal lesions. Besides the potential for acute complications, there is a significant recanalization risk. Surgical options include ligation, lobectomy, or pneumectomy [3].

Large AVMs can have profound hemodynamic consequences, whose severity increases with the size and complexity of the lesion. Right-to-left shunting results in rapid onset of cyanosis postnatally, and the resulting shunt can lead to high-output heart failure caused by the shunting of blood through the AVM whose medical management is challenging [8]. Prenatal left-to-right shunting through the patent ductus arteriosus may be an indicator of the hemodynamical significance of the AVM during fetal life, showcasing a volume and pressure overload to the left heart, as previously shown during the post-natal period [9].

Overall, high mortality has been reported in cases with prenatal diagnosis [8], possibly resulting from selection bias of more severe cases.

Akler et al. described a case diagnosed in a 16-week fetus, in which the parents opted for medical termination of the pregnancy due to poor prognosis. The first reported survival of a second-trimester diagnosis and posterior event-free survival was only described in 2013 [2]. As found in our case, the fetus presented retrograde flow across the ductus arteriosus and severe cardiomegaly; however, it was successfully managed with surgical removal of the fistula and pulmonary artery and left atria plasty.

## Conclusions

In conclusion, the diagnosis of large AVMs can have profound hemodynamic consequences, leading to challenges in medical management and high mortality rates, particularly in cases with prenatal diagnosis, often associated with complex management and difficult-to-determine prognosis. Our case report suggests that flow reversal through the ductus arteriosus could indicate a significant hemodynamic impact.

Our case report sheds light on the challenging clinical scenario of pulmonary AVMs, emphasizing their potential severity and the impact on fetal outcomes. It is crucial to recognize the gravity of early and severe presentations, where comprehensive counseling should be provided.
